# Supplementary material for: Direct Z-Scheme Heterojunction Catalysts Constructed by Graphitic-C3N4 and Photosensitive Metal-Organic Cages for Efficient Photocatalytic Hydrogen Evolution
Source: Nanomaterials (Basel). 2022 Mar 7;12(5):890. doi: 10.3390/nano12050890 (PMC8912648; doi:10.3390/nano12050890)
Supplement: Supplementary file 1 [file nanomaterials-12-00890-s001.zip › nanomaterials-1588978-supplementary.pdf]

nanomaterials

MDPI

Article

Direct Z-Scheme Heterojunction Catalysts Constructed by Graphitic-C_3N_4 and Photosensitive Metal-Organic Cages for Efficient Photocatalytic Hydrogen Evolution

Chuying Lv [1], Su Qin [2], Yang Lei [1], Xinao Li [1], Jianfeng Huang [1,*] and Junmin Liu [1,*]

[1] School of Materials Science and Engineering, The Key Laboratory of Low-Carbon Chemistry & Energy Conservation of Guangdong Province, Sun Yat-sen University, Guangzhou 510006, China; lvchy3@mail2.sysu.edu.cn (C.L.); leiy56@mail2.sysu.edu.cn (Y.L.); lixao@mail2.sysu.edu.cn (X.L.)
[2] School of Chemical Engineering and New Energy Materials, Zhuhai College of Science and Technology, Zhuhai 519041, China; qins3@mail2.sysu.edu.cn
* Correspondence: huangjf39@mail.sysu.edu.cn (J.H.); liujunm@mail.sysu.edu.cn (J.L.)

Table S1. The measured mass fractions of Pd and MOC-Q2 in g-C_3N_4/MOC-Q2 (0.3/0.7/1.0/2.0 wt%) catalysts.

Theoretical MOC-Q2 loading / wt%	Actual Pd loading / wt%	Actual MOC-Q2 loading / wt%
0.3	0.01	0.24
0.7	0.03	0.53
1	0.05	0.80
2	0.10	1.65

Mw (MOC-Q2) = 3452.82 and Mw (Pd) = 106.42.; MOC-Q2 wt% = Pd wt% / (212.84/3458.82).

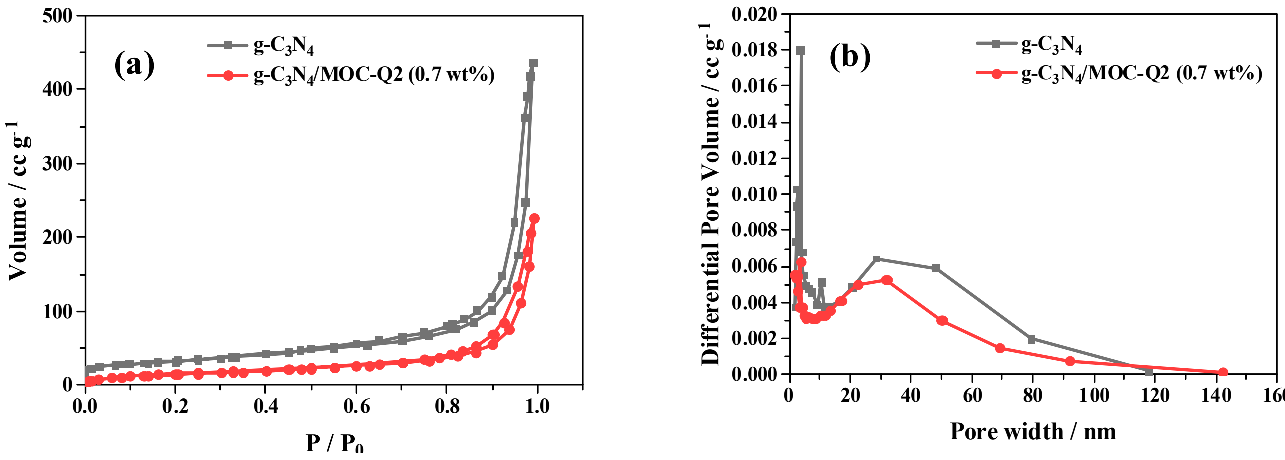

Figure S1. (**a**) N_2 adsorption-desorption isotherms and (**b**) pore size distributions of g-C_3N_4 and g-C_3N_4/MOC-Q2 (0.7 wt%).

Table S2. BET surface areas and pore volumes of g-C_3N_4 and g-C_3N_4/MOC-Q2 (0.7 wt%).

	g-C_3N_4	g-C_3N_4/MOC-Q2 (0.7 wt%)
BET surface area (m^2/g)	94.6	54.5
Total pore volume (cm^3/g)	0.47	0.33
Microporous pore volume (cm^3/g)	0.008	——
Mesoporous pore volume (cm^3/g)	0.47	0.33

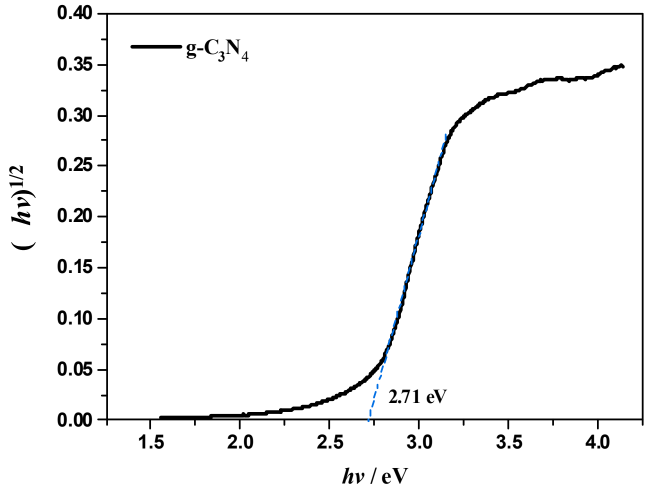

Figure S2. Tauc plot of the g-C_3N_4 sample.

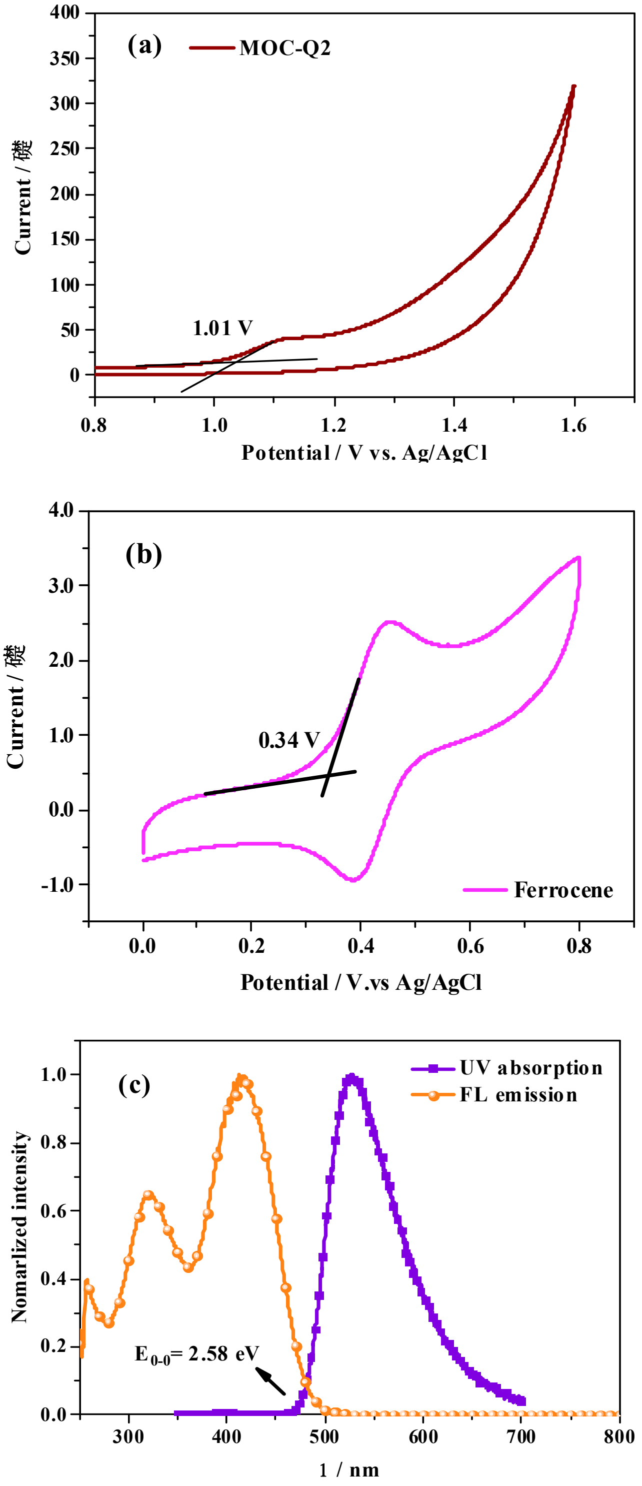

Figure S3. CV curves of (**a**) MOC-Q2 (0.1 mM) and (**b**) ferrocene in a mixed solvent of DMSO/CH_3CN (1:5 v/v) containing $(C_4H_9)_4NPF_6$ with a scan rate of 50 mV s^{-1}, and (**c**) normalized absorption and emission spectra of MOC-Q2.

Table S3. The oxidation potential, E_{0-0}, HOMO, and LUMO values of MOC-Q2.

Compound	E_{ox}/V vs. Ag/AgCl	E_{ox}/V vs. NHE	E_{0-0}/eV	HOMO/V vs. NHE	LUMO/V vs. NHE
MOC-Q2	1.01	0.97	2.58	0.97	–1.61

Table S4. Summary of the H_2 production amounts and the corresponding TONs within 5 h.

Material	H_2 yield/mmol/g[a]	$TON_{[Pd]}$[b]	$TON_{[MOC]}$[c]
MOC-Q2	4.28	7	15
Pd/g-C_3N_4/L-2 (0.7 wt%)	0.78	255	-
g-C_3N_4/MOC-Q2 (0.3 wt%)	13.47	9706	19,413
g-C_3N_4/MOC-Q2 (0.7 wt%)	32.11	10,478	20,955
g-C_3N_4/MOC-Q2 (1.0 wt%)	15.76	3407	6814
g-C_3N_4/MOC-Q2 (2.0 wt%)	14.76	1547	3094

[a] = H_2 yield in 5 h / total mass of catalysts;. [b] = H_2 yield vs. Pd loading amount = [a]× 0.001 × 106.42[d] / corresponding Pd loading mass fraction; [c] = H_2 yield vs. MOC loading amount = [a] × 0.001 × 3458.82[e] / corresponding MOC loading mass fraction; [d]: Relative molecular mass of Pd; [e]: Relative molecular mass of MOC-Q2

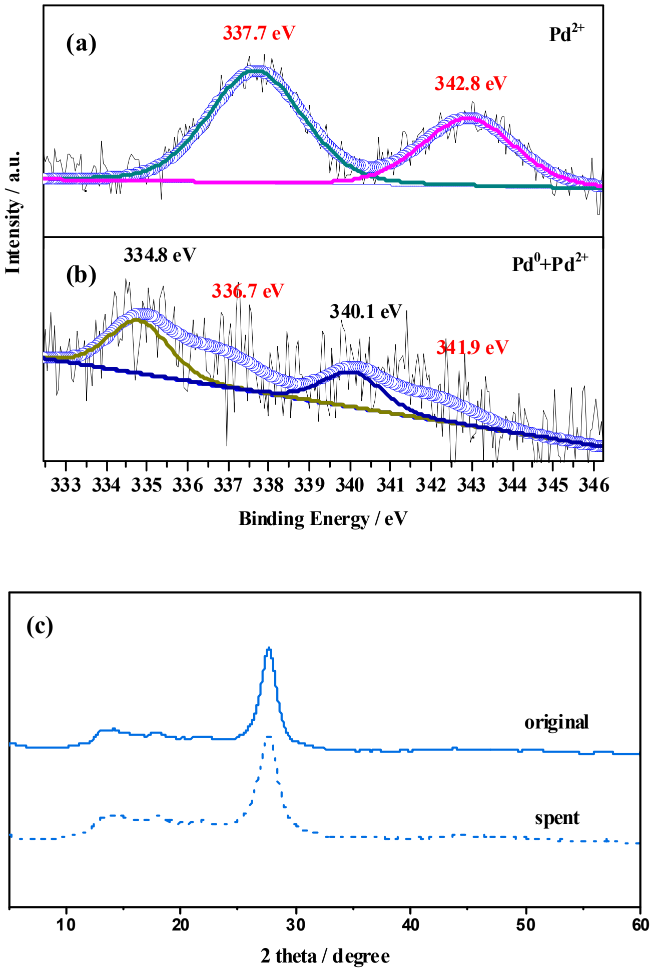

Figure S4. The XPS Pd 3d spectra of g-C_3N_4/MOC-Q2 (2 wt%) (**a**) before and (**b**) after 10 h photocatalysis, and (**c**) the XRD patterns of g-C_3N_4/MOC-Q2 (2 wt%) samples before and after photocatalytic reaction.

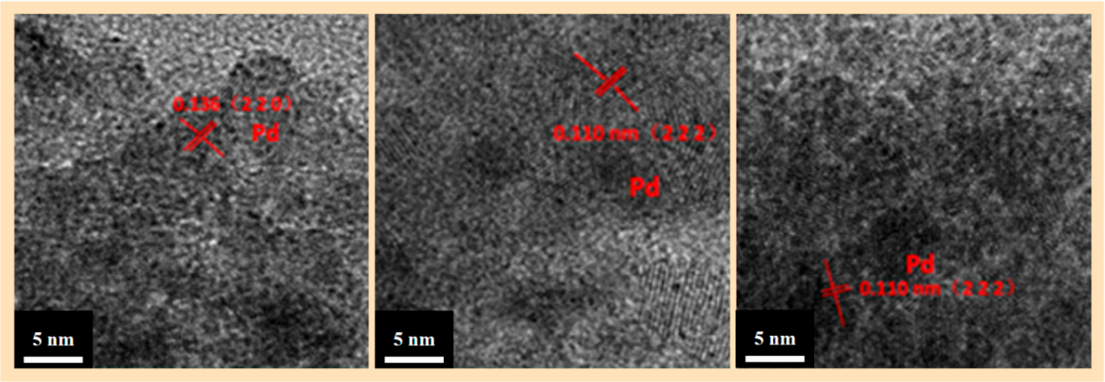

Figure S5. High-resolution TEM images of the spent g-C3N4/MOC-Q2 (2 wt%) sample after photocatalytic reaction.

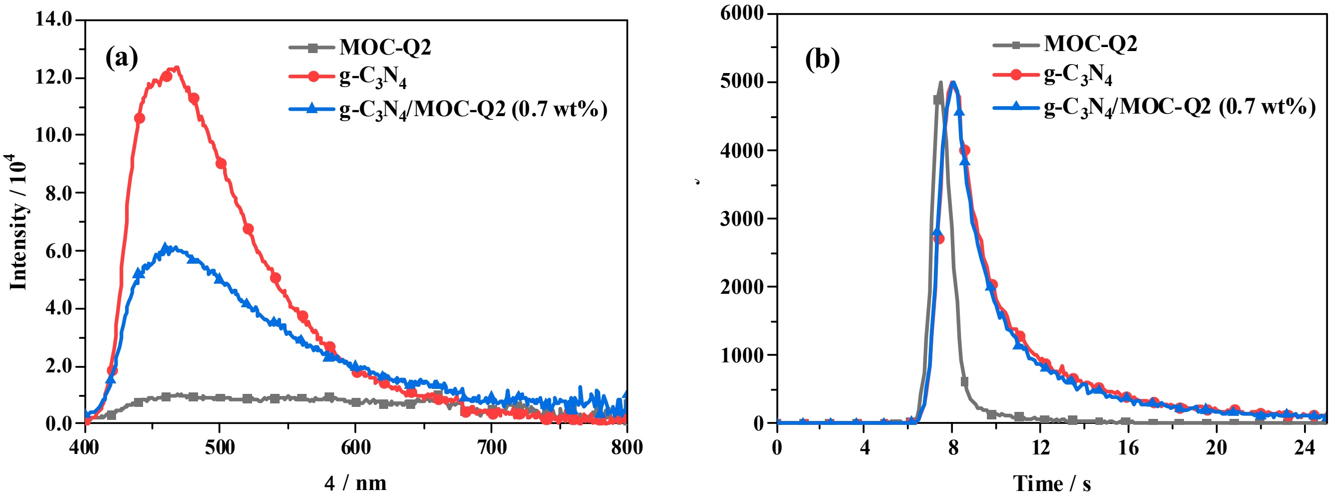

Figure S6. (**a**) Steady state and (**b**) time-resolved PL spectra of MOC-Q2, g-C3N4, and g-C3N4/MOC-Q2 (0.7 wt%).
